# Supplementary figures and images for: ORF48 is required for optimal lytic replication of Kaposi’s sarcoma-associated herpesvirus
Source: PLoS Pathog. 2024 Aug 26;20(8):e1012081. doi: 10.1371/journal.ppat.1012081 (PMC11379392; doi:10.1371/journal.ppat.1012081)

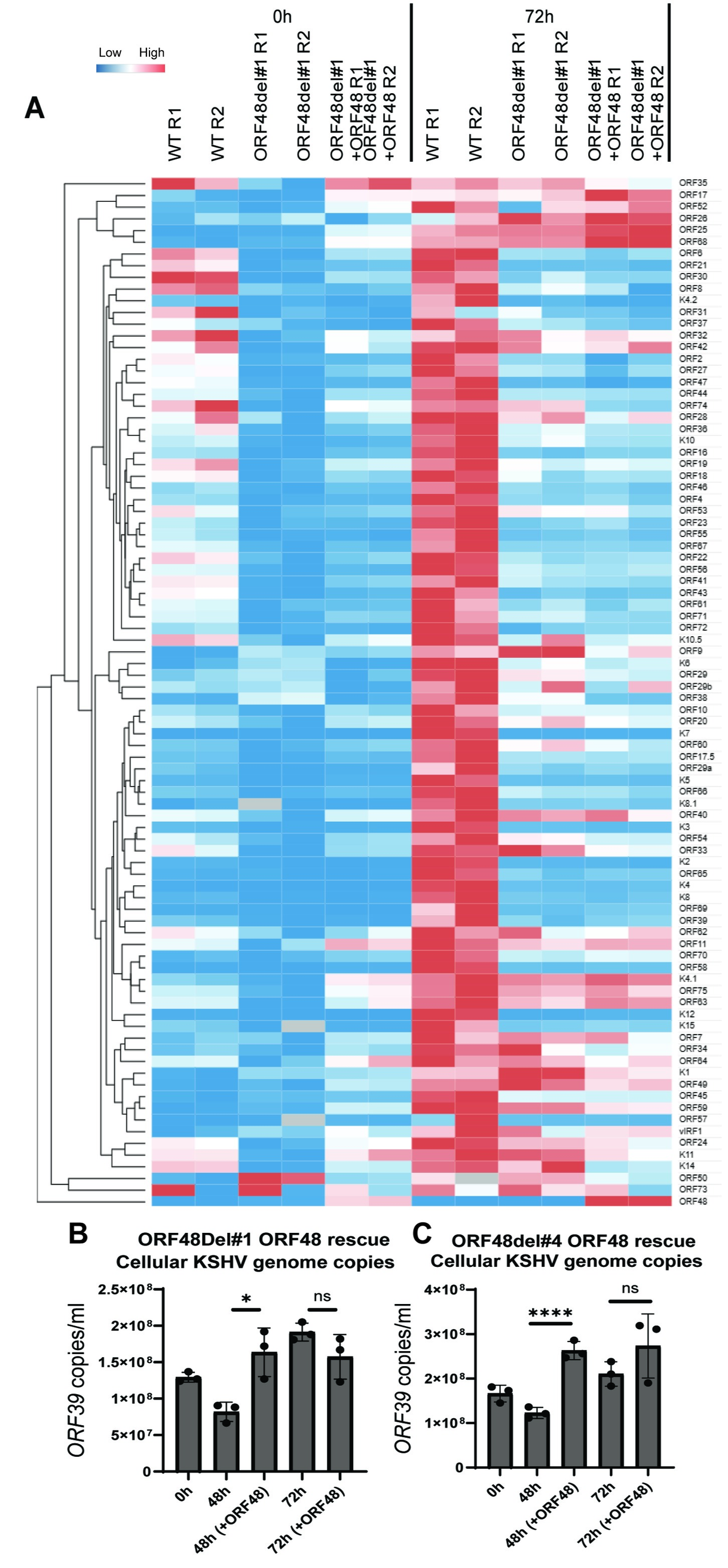

Supplement: S1 Fig — (A) The experiments were performed in the following 3 groups: 1. iSLK.BAC16 WT + vector; 2. iSLK.BAC16 ORF48del#1 + vector; 3. iSLK.BAC16 ORF48del#1 + pORF48-STREP. They were either treated with Dox for 72 hours or not. The KSHV array was performed. Higher transcript expression levels are indicated by red and lower expression levels by blue as shown in the key. (B)(C) Cellular KSHV genome copies were quantitated using a genomic primer based on the ORF39 coding sequence as previously described. A STREP-tagged ORF39 [37] was used to generate the standard curve. Data are presented as mean ± s.d. from at least three independent experiments. *indicates p<0.05. ** indicates p<0.01 *** indicates p<0.001 **** indicates p<0.0001 by Student’s t-test. (TIF) [file ppat.1012081.s001.tif]

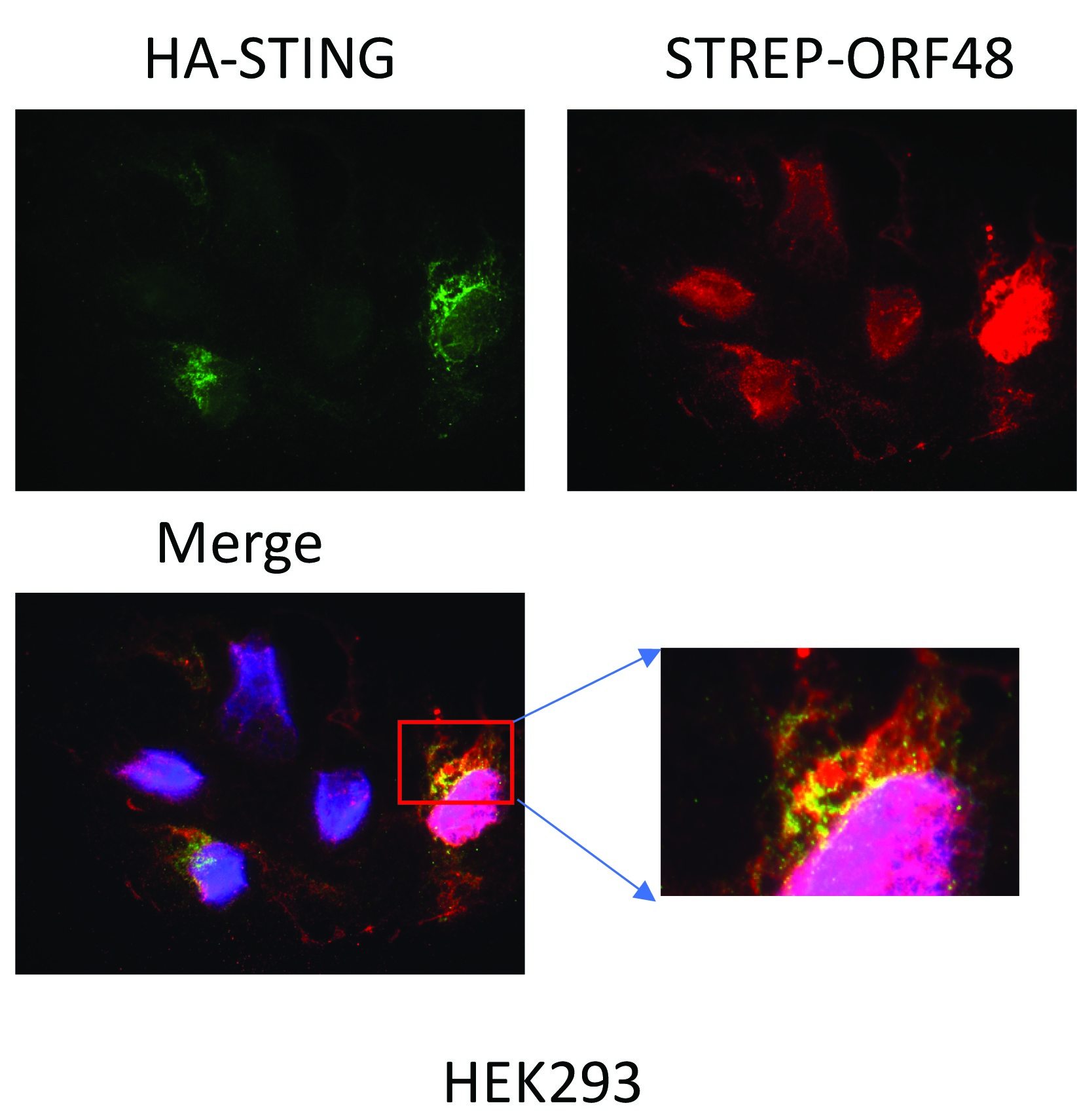

Supplement: S2 Fig — HEK293 cells were transfected with STREP-tagged-ORF48 and HA-STING. Forty-eight hours later, cells were stained with mouse HA (Green), rabbit STREP (red), and DAPI (Blue). (TIF) [file ppat.1012081.s002.tif]

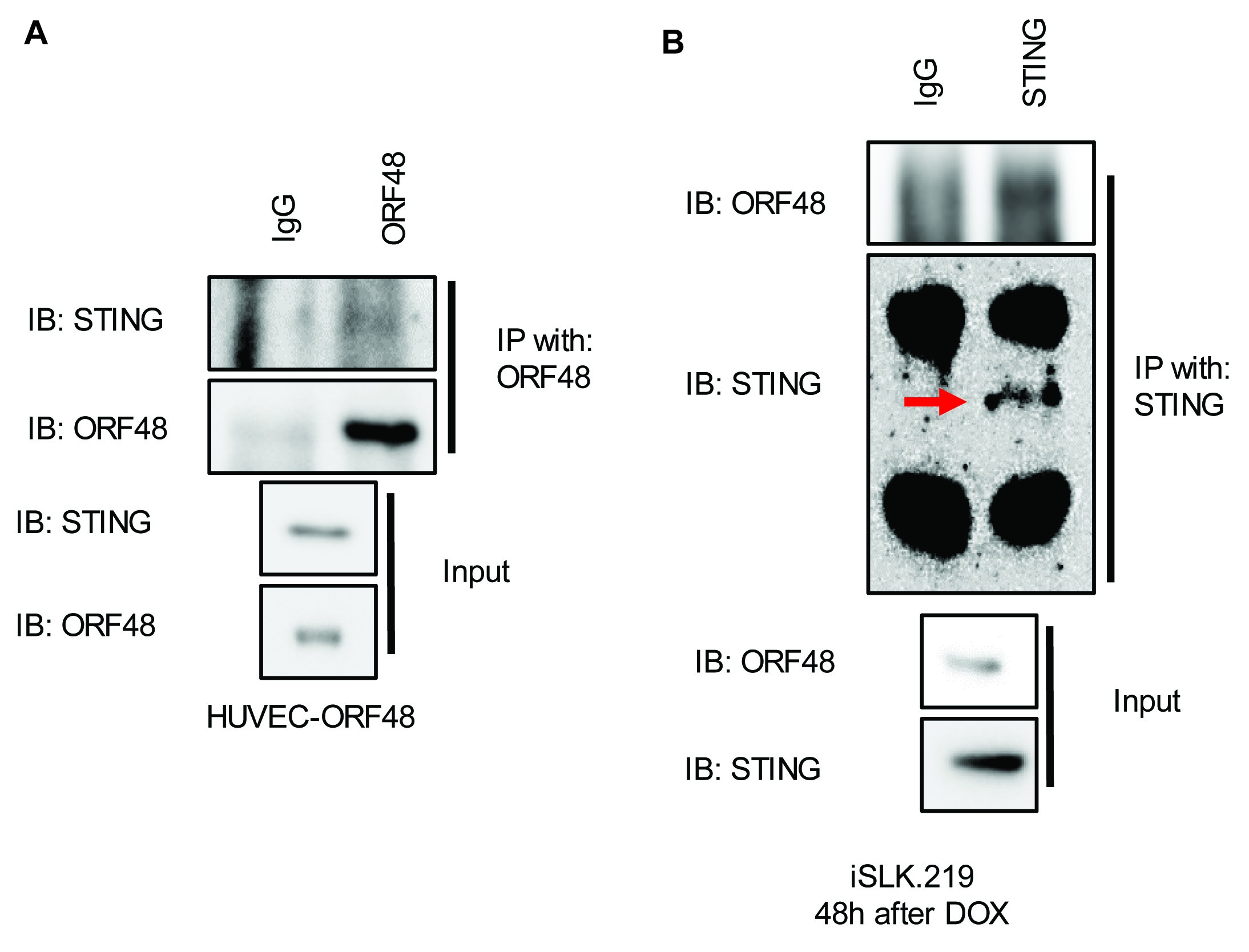

Supplement: S3 Fig — (A) Co-immunoprecipitation of endogenous STING and FLAG-ORF48. HUVEC-ORF48 cell lysates were immunoprecipitated with either rabbit IgG or rabbit ORF48 antibody and protein A/G beads. STING or ORF48 antibodies and light-chain-specific secondary antibodies were used for band detection. (B) Co-immunoprecipitation of endogenous STING and ORF48. iSLK.219 cells were treated with Dox for 48 hours and cell lysates were immunoprecipitated with either rabbit IgG or STING antibody and protein A/G beads. STING or ORF48 antibodies were used for band detection. (TIF) [file ppat.1012081.s003.tif]

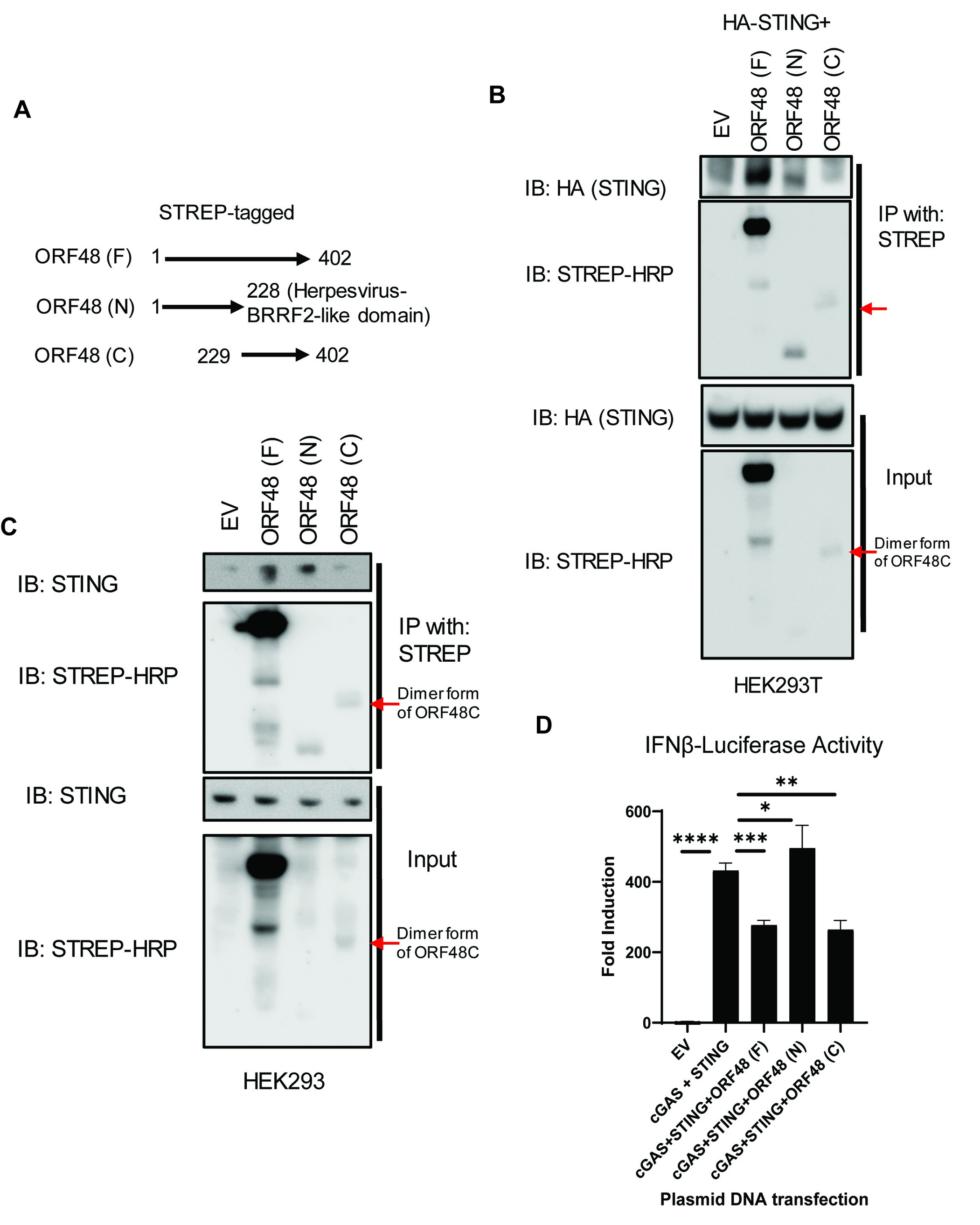

Supplement: S4 Fig — (A) Diagram of ORF48 (F), ORF48 (N, 1–228), and ORF48 (C, 229–402). (B) Co-immunoprecipitation of HA-STING and STREP-ORF48 (F, N, and C). HEK293T cells were transfected with HA-STING, an empty backbone, and STREP-ORF48 variants as shown. Forty-eight hours later, cell lysates were immunoprecipitated with STREP antibody and protein A/G beads. HA or STREP antibodies were used for band detection. We repeatedly detected a specific band at about 35 kDa using multiple sequencing-validated clones of ORF48C, projected as the dimmer form of ORF48c. (C) Co-immunoprecipitation of endogenous STING and STREP-ORF48 variants. HEK293 cells were transfected with an empty backbone or STREP-ORF48 as shown. Forty-eight hours later, cell lysates were immunoprecipitated with STREP antibody and protein A/G beads. STING or STREP antibodies were used for band detection. (D) IFNβ-luc assay in HEK293 cells. Per well in 24 well plates, the following plasmids were transfected, cGAS (100ng), STING (5ng), IFNβ-luc (100ng), CMV-Renilla (10ng), ORF48 variants or EV (200ng). Forty-eight hours later, cells were harvested, lysed, and subjected to a Dual-luciferase assay. Firefly/Renilla ratios were generated in each group and all groups were then normalized to their EV control group respectively to generate fold induction. Data are presented as mean ± s.d. from at least three independent experiments. *indicates p<0.05. ** indicates p<0.01 *** indicates p<0.001 **** indicates p<0.0001 by Student’s t-test. (TIF) [file ppat.1012081.s004.tif]

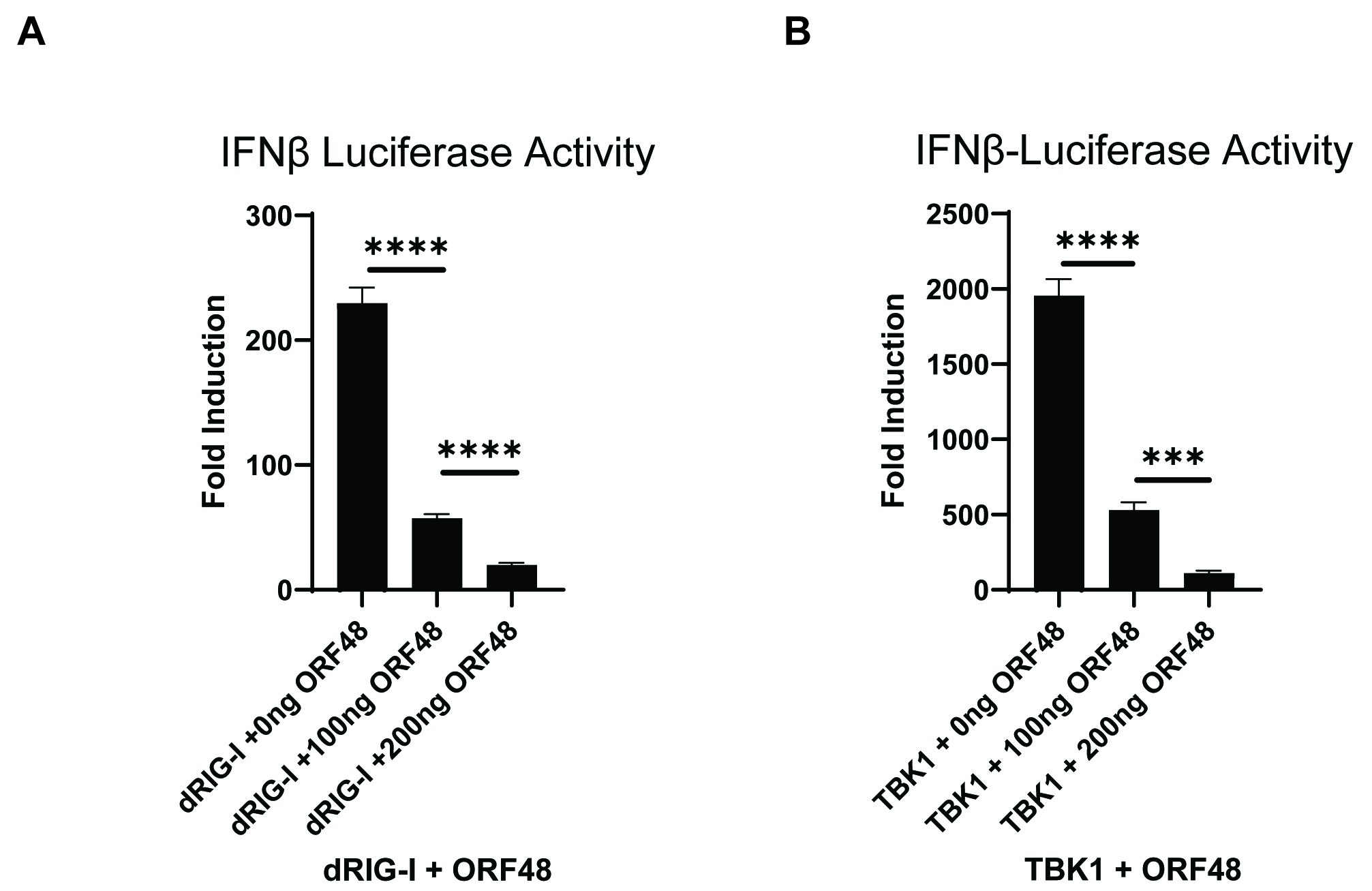

Supplement: S5 Fig — Per well in 24 well plates, the following plasmids were transfected, (A) dRIG-I (100ng) or (B) TBK1 (100ng), IFNβ-luc (100ng), CMV-Renilla (10ng), ORF48 (0, 100ng or 200ng). Forty-eight hours later, cells were harvested, lysed, and subjected to a Dual-luciferase assay. Firefly/Renilla ratios were generated in each group and all groups were then normalized to their EV control group respectively to generate fold induction. Data are presented as mean ± s.d. from at least three independent experiments. *indicates p<0.05. ** indicates p<0.01 *** indicates p<0.001 **** indicates p<0.0001 by Student’s t-test. (TIF) [file ppat.1012081.s005.tif]
